# Indoor Thermal Environments, Cooling Access, and Energy Burden in New Orleans, LA: Challenges and Opportunities for Heat Adaptation

**DOI:** 10.1007/s11524-026-01096-w

**Published:** 2026-06-10

**Authors:** Lena Easton-Calabria, Caroline Reed, Teague Ruder, Jordan Mychal, Jacopo Scazzosi, Ramya Chari, Brian Vant-Hull, Julia Kumari Drapkin, Jaime Madrigano

**Affiliations:** 1 RAND, Arlington, VA USA; 2 University of Oxford, Oxford, UK; 3 ISeeChange, Inc., New Orleans, LA USA; 4 RAND, Boston, MA USA; 5 The City College of New York, New York, NY USA; 6 Johns Hopkins Bloomberg School of Public Health, 615 N. Wolfe Street, Baltimore, MD 21205 USA

**Keywords:** Heat, Vulnerability, Adaptation, Housing, Air conditioning, Energy

## Abstract

Extreme heat is a significant and growing health hazard in urban locations around the world, particularly in the Southeastern United States (U.S.). While most extreme heat research and interventions are focused on ambient outdoor conditions and the neighborhood environment, the indoor residential environment is where the most severe heat-health consequences occur. The aim of this study was to characterize the indoor thermal environments and identify predictors of high indoor temperatures for residents of heat-vulnerable neighborhoods within New Orleans, Louisiana, within the context of current heat adaptation measures and issues of energy insecurity. We conducted surveys with both open- and closed-ended questions and measured indoor temperature over 2-week sampling periods in 114 households across two heat vulnerable New Orleans wards during the warm seasons of 2023 and 2024. Our study found that a combination of AC type and use, along with outdoor daily maximum temperature, were significant predictors of indoor maximum overnight temperature. Our results indicate that households without AC, using window AC units, or those not running central AC all or most of the time struggled to maintain 80 degrees Fahrenheit overnight (a threshold deemed appropriate by a recent healthy homes ordinance) once outdoor daily maximum temperatures exceeded 90 degrees Fahrenheit. Homeownership, compared to renting, was associated with higher overnight indoor temperatures, greater variability in typical AC use patterns, and greater sensitivity of summer monthly energy expenditures to differences in AC use patterns, potentially indicating that this group is practicing energy limiting behavior. This paper contributes to limited literature on indoor thermal environments, particularly in the Southeastern U.S., and underscores the importance of housing and energy burden in heat adaptation.

**Supplementary Information:**

The online version contains supplementary material available at 10.1007/s11524-026-01096-w.

## Introduction

Extreme heat is a critical and growing health hazard around the world. Globally, July 2025 was the third warmest July on record behind only July 2023 and 2024 [1]. In the U.S., heat is responsible for more fatalities than any other type of weather event [2]. Furthermore, these impacts are not distributed equally, and low-income and historically marginalized populations are among those most at-risk for heat-related illness and mortality [3, 4]. Due to historic and ongoing discriminatory structures and policies, these populations often live in neighborhoods with lower housing quality and higher temperatures, with the latter a result of well-documented disparities in tree canopy and vegetative cover and an abundance of asphalt and concrete [5–7]. What is less characterized is the indoor residential environment for populations at high risk for heat-related illness.

While most extreme heat research and interventions are focused on the outdoor thermal environment, the indoor residential environment is where the most severe heat-health consequences occur. Heat kills more people inside their homes than anywhere else [8]. For example, almost half of all heat-related deaths in 2023 in Louisiana occurred in the decedent’s home [9]. In the U.S., people spend approximately 90% of their time in indoor environments and the indoor residential environment is frequently the “first line of defense” when it comes to climate-sensitive health impacts [10, 11]. Given this, more research on the indoor thermal environment – and indoor *residential* environment, in particular – is needed to improve health outcomes during extreme heat events. This is especially important as time spent at home has increased among American adults over the last two decades [12].

Existing evidence indicates that access to residential air conditioning (AC) is the single biggest factor associated with a decline in heat-related mortality rates in the U.S. over the twentieth century [13]. However, even in parts of the U.S. where AC penetrance is high, populations that are the most heat-sensitive, including the poor and elderly on fixed incomes, often lack access to AC [14]. Others experience “energy poverty,” whose accepted definitions include a household’s inability to access adequate energy services due to financial constraints [15], spending more than 10% of one’s income on energy costs [16], and, more generally, limited resources for energy usage for heating, cooling, or other medically-necessary expenses.

In part due to the disconnect between the location of commonly employed interventions (outdoor neighborhood environment) and where fatal heat exposure occurs (indoor environment), as well as the well-known problems of energy insecurity and poverty among populations most at risk for heat illness and death, some policymakers have begun to target new interventions for the indoor residential environment. As part of this effort, municipalities around the country, including New Orleans, Louisiana, are beginning to implement indoor residential cooling standards [17]. However, as is the case in most cities that have implemented such ordinances, the feasibility, enforceability, and the impact of these standards remains unknown.

This paper aims to characterize the indoor residential thermal environment and identify predictors of high indoor temperatures in a sample of owner- and renter-occupied units from residents of multiple low-income, predominantly Black neighborhoods across two wards in New Orleans, Louisiana. We examine the relationship between environmental and residential characteristics and the indoor thermal environment, with attention to issues of energy insecurity and in response to the need for further research on heat exposure within underserved communities. Specifically, we focus our analysis on predictors of *overnight* indoor thermal environments because this is the time period when study participants were most likely to be inside their residence–and given that existing research has shown that high overnight temperatures contribute to excess mortality [19].

Data, results, and implications for policy, specifically indoor residential cooling standards, and future research areas are discussed. Results are contextualized within statistically downscaled temperature projections for New Orleans over the next fifty years (2025–2075) under multiple emissions scenarios (SSP2.4–5, SSP3.7–0, and SSP5.8–5) in order to understand how current temperature challenges are likely to grow in the future. Our focus on New Orleans is particularly relevant as it is in the top five large U.S. cities where trends have increased for three of four aspects of heat waves (timing, frequency, and duration) [20], along with a rise in heat-related deaths [21]. Furthermore, the southern U.S. is projected to experience the most significant impacts from heat-related mortality in the country through 2100 across a set of possible future climate scenarios [22]. Given this, a better understanding of implementation issues for the current standard and other interventions targeting heat morbidity and mortality is vital.

## Methods

### Study Design and Data Collection

Neighborhoods across two wards in New Orleans – Hollygrove/Hollygrove-Dixon (warm season of 2024) and the Florida, Bunnyfriend, and Desire areas (warm season of 2023) – were selected for this study due to a multitude of factors, including tree canopy and impervious pavement coverage [23], historical redlining [24], income level, and other urban heat risk factors [9]. These neighborhoods share many characteristics with one another. For example, they are majority Black and have lower household incomes and higher rates of poverty than the average in New Orleans and the United States more broadly. Most households are either single, female-headed households with children or adult households without children. Although significant gains in educational attainment have been made since 2000, a significant portion of both renters and owners pay over 30% of their income on housing [25].

Participants were recruited through a combination of community-based and digital outreach strategies. A large portion of study participants were recruited through in-person events in collaboration with neighborhood organizations. Recruitment was amplified by email notifications to the ISeeChange [26] digital community, and through social media. Community partners played an essential role in promoting the opportunity, circulating information both in meetings and through digital channels.

Participants were onboarded through an in-person intake interview as part of a larger study on heat and health in New Orleans (Supplementary Fig 2). During the interview, participants were provided an overview of the study, informed consent was obtained, and comprehensive participant data was collected through a baseline survey. The survey was administered using Android tablets programmed with Open Data Kit (ODK) [27], an open-source mobile data collection platform. The use of ODK and portable tablets facilitated survey deployment at participant homes, which in turn supported recruitment and reduced participant burden. Survey questions covered a wide variety of factors that can influence exposure to heat and enhance vulnerability to heat risk, including demographic and socioeconomic information, characteristics of the residential environment, access to cooling, and self-reported home energy costs. In a subset of participants, we verified the self-reported home energy costs with copies of their utility bills.

The intake survey was followed by a two-week sampling period during which we continuously monitored temperature and humidity inside participant bedrooms using Verizon Critical Asset Sensors (CAS) [28]. Three sampling periods (or “study cohorts”) were conducted each year, resulting in three 2-week data collection periods in the Florida, Bunnyfriend, and Desire areas in 2023 (cohorts 1–3) and three 2-week data collection periods in the Hollygrove/Hollygrove-Dixon areas in 2024 (cohorts 4–6). CAS provides real time, granular temperature and humidity readings every 15 min. To capture sleeping temperature experience, sensors were placed away from windows, A/C units, and at “waist to chest” levels. Results were monitored daily, and abnormal readings were investigated by the field team to ensure that sensors were accurately capturing room temperature. The “normal” range was defined as 60 F < T < 105 F; outside of this range, we considered that a sensor was likely malfunctioning, had been accidentally placed in front of an AC vent, or that there was another issue and needed to be investigated and possibly replaced. We conducted a zeroing procedure to estimate and correct systematic differences between the readings from the sensors. Over the course of the study, data was collected from each sensor approximately every 15 min over six, two-week sample periods between June and early October in 2023 and 2024. This sampling period is consistent with prior work characterizing the Louisiana warm season as April through October [9]. Hourly temperature averages were calculated as the mean of all the readings that were recorded during that hour for all hours that had at least three readings. Maximum and mean indoor overnight temperatures were calculated by taking the maximum and average, respectively, hourly temperature readings from participants’ bedrooms during the period of 7 pm to 7 am.

Outdoor ambient temperature and humidity data were obtained from the virtual platform Weatherwise, which combines data from weather instruments, agricultural probes, and other sensors stationed throughout New Orleans through the Weatherwise partnership between NOLA Ready and WeatherSTEM. We took the average of readings from four of the 26 outdoor stations, selected based on proximity and similarity to the neighborhoods from where our participants were drawn.

### Statistical Analysis

Descriptive statistics were computed for participant information, residential characteristics, and meteorological data to provide a comprehensive overview of data results. Because study participants may have elected to take part in up to three (2-week) periods of data collection, we also described participant and residential characteristics by the amount of contributed person-time (days) in our analytic data set. Since time lags of anywhere between 3 h and 2 days between outdoor and indoor temperatures have been observed in prior literature [29, 30], we calculated Spearman correlation coefficients between maximum overnight indoor temperature and daily maximum outdoor temperature of the preceding day across all person-days. Because installing and replacing sensors could result in dramatic changes to hourly readings that were not representative of usual conditions inside the home, we conducted an outlier analysis and removed any hourly observation that was more than four standard deviations from the mean. Self-reported home energy costs, provided in response to being asked what participants’ monthly energy costs average during the summer, were generally similar to costs reported from energy bills in the subset of participants where utility bills were made available. Therefore, we used self-reported home energy costs (available for our entire sample) in our models.

Linear mixed effects regression models were employed to model predictors of maximum indoor overnight temperature. Maximum indoor overnight temperature was chosen to facilitate policy relevance of our findings, since the Healthy Homes ordinance is based on a maximum bedroom temperature. However, in sensitivity analyses, we also examined predictors of mean indoor overnight temperature which may provide a better representation of the overall overnight conditions. We included the following independent variables in the models, in part, due to their association with indoor thermal conditions in previous literature [29, 31, 32]: maximum daily ambient (outdoor, preceding day) temperature: participant age, race, gender, and household income; an indicator for combined type and use of AC (households running central AC all or most of the time during hot weather versus households with no AC, window units, or those not running central AC all or most of the time during hot weather), home type, home material, home ownership status, self-report of trees providing shade to residence, self-reported monthly energy costs during the summer, floor of bedroom, an indicator for use and type (ceiling/portable) of fans, other methods of indoor temperature adjustment (e.g., window blinds, window/door adjustment, etc.), and study cohort. The model included a random intercept for participants to account for repeated measures. Because outdoor temperature and AC type and use were the strongest independent predictors of indoor temperature, in a second model, we included an interaction term between maximum daily ambient (outdoor, preceding day) temperature and type/use of AC. To facilitate interpretation of how type/use of AC modifies the relationship between daily maximum outdoor temperature and maximum indoor overnight temperature, we plotted the predicted values from a regression model including this interaction term and adjusted for selected covariates.

Finally, we examined the distribution of self-reported home energy costs according to type and use of AC visually through boxplots. We conducted a two-way ANOVA test to determine if there was a difference in mean household reported summer monthly energy costs by reported type and use of AC and across homeowner and renter status. All statistical analysis was conducted in R, version 4.4.1.

### Climate Projections

To contextualize our results within a changing climate, we used Localized Constructed Analogs 2 (LOCA2) [33], a statistically downscaled climate data product available at 6 km resolution in the continental U.S. It includes 27 climate models available in the Coupled Model Intercomparison Project Phase 6 (CMIP6) [34]. LOCA2 was used to project days with a high above 90 degrees Fahrenheit (ambient air temperature) in New Orleans over the next fifty years (2025–2075) under medium (SSP2-4.5), high (SSP3-70), and very high (SSP5-8.5) emissions scenarios.

## Results

Across both neighborhoods and over two sampling years, 127 residents were recruited to participate in our study. However, n = 13 participants chose not to participate in our ongoing data collection activities, resulting in a total sample size of n = 114 residents, contributing 1,604 person-days of follow-up, for the present study. On average, residents participated for one 2-week cohort period, though some study participants elected to take part in more than one sampling period, resulting in a maximum of 30 person-days from a single participant (IQR: 10–16 days). Participants in our study were predominantly Black or African American and from low-income households living under the city’s median household income level of $55,580 (Table 1). Women made up a majority of the sample, although a greater proportion of men participated in Year 2 of the study. Approximately half of the sample reported renting their residence and half were homeowners. Almost all of the sample reported having access to AC in their residence (Table 1), although reported use varied. Just under 90% of participants reported running their AC always or most of the time during hot weather, while the rest reported running it half of the time or less. Approximately ¾ of the sample reported using central AC, while the remainder used window units.

**Table 1:** Population and residential characteristics, NOLA HEAT-MAP Study, 2023–2024

Characteristic, %		Year 1 (n = 64)	Year 2 (n = 50)	Total (n = 114)
Race	Black or African American	84.4%	88%	86%
	Hispanic/Latino	1.6%	0	0.9%
	American Indian or Alaska Native	1.6%	0	0.9%
	White	9.4%	6%	7.9%
	Multiracial	1.6%	2%	1.8%
	Other	1.6%	0	0.9%
	Prefer not to say	0	4%	1.8%
Gender	Woman	75%	60%	68.4%
	Man	21.9%	36%	28.1%
	Non-binary	1.6%	0	0.9%
	Prefer not to say	1.6%	4%	2.7%
Age	18–29	9.4%	6.2%	8%
	30–41	31.2%	14.6%	24.1%
	42–53	25%	14.6%	20.5%
	54–65	23.4%	20.8%	22.3%
	66–77	10.9%	33.3%	20.5%
	78–85	0	10.4%	4.5%
Annual household income	$0-$14,999	47.5%	46.8%	47.2%
	$15,000-$44,999	32.2%	36.2%	34%
	$45,000-$89,999	20.3%	12.8%	17%
	$90,000 +	0	4.3%	1.9%
Education	Less than a high school degree	23.4%	16%	20.2%
	High school degree	26.6%	36%	30.7%
	Some college	25%	16%	21.2%
	Associate degree	7.8%	6%	7.0%
	Bachelor's degree	10.9%	10%	10.5%
	Master’s degree	0	2%	9.6%
Home status	Rental	57.8%	44%	51.8%
	Own/family owns home	42.2%	56%	48.2%
Home type	Single-family home	59.4%	58%	58.8%
	Duplex/Double-family home (New Orleans shotgun or Camelback)	23.4%	26%	24.6%
	Apartment/multi-unit housing	10.9%	14%	12.3%
	Other	6.2%	2%	4.4%
Home material	Wood	57.8%	52%	55.3%
	Concrete	7.8%	6%	7%
	Brick	6.2%	6%	6.1%
	Multiple/Unknown	28.1%	36%	31.6%
AC type	Window	25%	28%	26.3%
	Central	73.4%	72%	72.8%
	None	1.6%	0	0.9%
Total # of building/home floors	Zero	10.9%	6%	8.8%
	One	54.7%	64%	58.8%
	Two	31.2%	20%	26.3%
	Three	0	6%	2.6%
	Five	1.6%	4%	2.6%
	Six	1.6%	0	0.9%
Floor that participant sleeps on	Zero	4.7%	6%	5.3%
	One	67.2%	80%	72.8%
	Two	28.1%	12%	21.1%
	Three	0	2%	0.9%
Use of fans	None	4.7%	22%	12.3%
	Ceiling	39.1%	34%	36.8%
	Portable	17.2%	26%	21.1%
	Multiple	39.1%	22%	29.8%
Use of other means of cooling	Adjust blinds	48.4%	38%	43.9%
	Open doors	4.7%	4%	4.4%
	Open windows	1.6%	2%	1.8%
	Other items	4.7%	16%	9.6%
	Multiple	40.6%	40%	40.4%
Self-reported shade from trees	Yes	15.6%	24%	19.3%
	No	76.6%	60%	69.3%
	Partial	7.8%	16%	11.4%
Receiving energy assistance	Yes	25%	28%	26.3%
	No	71.9%	72%	71.9%
	Unsure	3.1%	0	1.8%

Maximum overnight temperatures varied across participants and time and ranged between 65.9—101 degrees Fahrenheit over the study period. Across all person-days, there was a greater proportion of observations from homes with window AC units (42%) or no A/C (4%) in the highest quartile of maximum overnight indoor temperatures compared with the lowest quartile of maximum overnight indoor temperatures, where the prevalence was 12% and 0%, respectively (Supplemental Table 1). The lowest quartile of maximum overnight indoor temperatures was also made up primarily of observations from participants who rented their home (65%) versus homeowners, whereas the highest quartile of maximum overnight temperatures was primarily observed in residences owned by the participant (62%). There was a weak, positive correlation between maximum indoor overnight temperature and the preceding day’s daily maximum outdoor temperature that varied by study cohort, with greater correlation seen in Year 2 (Hollygrove/Hollygrove-Dixon neighborhood) of our study (Supplementary Fig. 1).

In a linear mixed effects model, we found a few statistically significant predictors of daily maximum indoor overnight temperature (Table 2). For every degree increase in the preceding day’s daily maximum outdoor temperature, maximum indoor overnight temperature increased by 0.22 degrees (95% CI: 0.18, 0.27). No AC, using window AC, including all or most of the time, or limiting use of central AC (i.e., running it only half of the time or less) increased indoor temperature by 3.49 degrees (95% CI: 1.13, 5.86), compared to running central AC all or most of the time. There were observed differences in the maximum indoor overnight temperature by study cohort, with greater maximum indoor overnight temperatures observed in cohort 3, compared to cohort 1. Finally, there was a marginal association with some predictors, including self-reported monthly energy costs and homeownership status. Daily maximum overnight indoor temperature decreased by 0.01 degrees (95% CI: −0.01, 0.0) for every dollar increase in energy costs. Home ownership (versus renting) also increased maximum overnight indoor temperature by 2.21 degrees (95% CI: −0.38, 4.81). Portable fan usage increased maximum indoor overnight temperature by 3.36 degrees (95% CI: −0.31, 7.03), compared to no fan usage.

**Table 2:** Mean difference (and 95% confidence interval) in daily overnight maximum indoor temperature associated with a one-unit increase of or in comparison to a reference value for independent variables*

	Estimate	95% CI		p-value
Daily maximum outdoor temperature (per 1 °F)	0.22	0.18,	0.27	** < 0.0001**
Air conditioning type and use				
Central AC, All/Most Time	Reference			
No AC, Any Window AC, Central AC Half Time/Rarely	3.49	1.13,	5.86	**0.004**
Race				
All other races	Reference			
Black race	−0.49	−4.49	3.51	0.809
Gender				
Female	Reference			
Male	1.47	−0.94	3.88	0.227
Non-binary	−6.68	−18.28	4.93	0.256
Age (per 1 year)	−0.02	−0.10	0.05	0.535
Household income				
$0-$14,999	Reference			
$15,000 -$44,999	−0.18	−2.51	2.14	0.875
$45,000—$89,999	−2.78	−6.25	0.69	0.115
$90,000 +	−0.22	−7.42	6.98	0.951
Refused to answer	−0.14	−4.07	3.79	0.944
Home type				
Single	Reference			
Apartment/multi unit	1.83	−1.37	5.03	0.258
Duplex/Double	−0.48	−3.11	2.16	0.719
Other	0.72	−4.10	5.54	0.767
Home material				
Brick	Reference			
Concrete	−3.14	−8.37	2.09	0.236
Multiple/Unknown	−0.32	−4.61	3.97	0.882
Wood	−1.06	−5.03	2.92	0.598
Study cohort				
1	Reference			
2	−0.12	−0.68	0.43	0.670
3	3.00	0.49	5.50	**0.020**
4	−0.96	−3.95	2.03	0.523
5	−0.43	−3.69	2.82	0.793
6	−0.18	−4.43	4.07	0.933
Self-reported energy costs (per $)	−0.01	−0.01	0.00	0.063
Self-reported trees provide shade to residence				
No	Reference			
Yes	1.13	−1.05	3.31	0.305
Homeownership				
Renter	Reference			
Owns home	2.21	−0.38	4.81	0.094
Use of fans				
None	Reference			
Ceiling	1.74	−2.00	5.48	0.357
Multiple	2.81	−1.24	6.86	0.172
Portable	3.36	−0.31	7.03	0.072
Use of other means of cooling				
Multiple	Reference			
Blinds	−0.25	−2.51	2.00	0.823
Open doors	−0.37	−5.60	4.87	0.889
Other items	−1.70	−5.13	1.74	0.328
Open window	−0.11	−6.89	6.66	0.974
Floor that participant sleeps on (per level)	−0.38	−2.32	1.56	0.695

^*^Models included a random intercept for participant; N = 109, with 1,532 person-days of observation. Three participants were missing information on gender and 2 participants were missing information on homeownership status. Two person-days were excluded because of missing outdoor temperature values

**Fig. 1:**
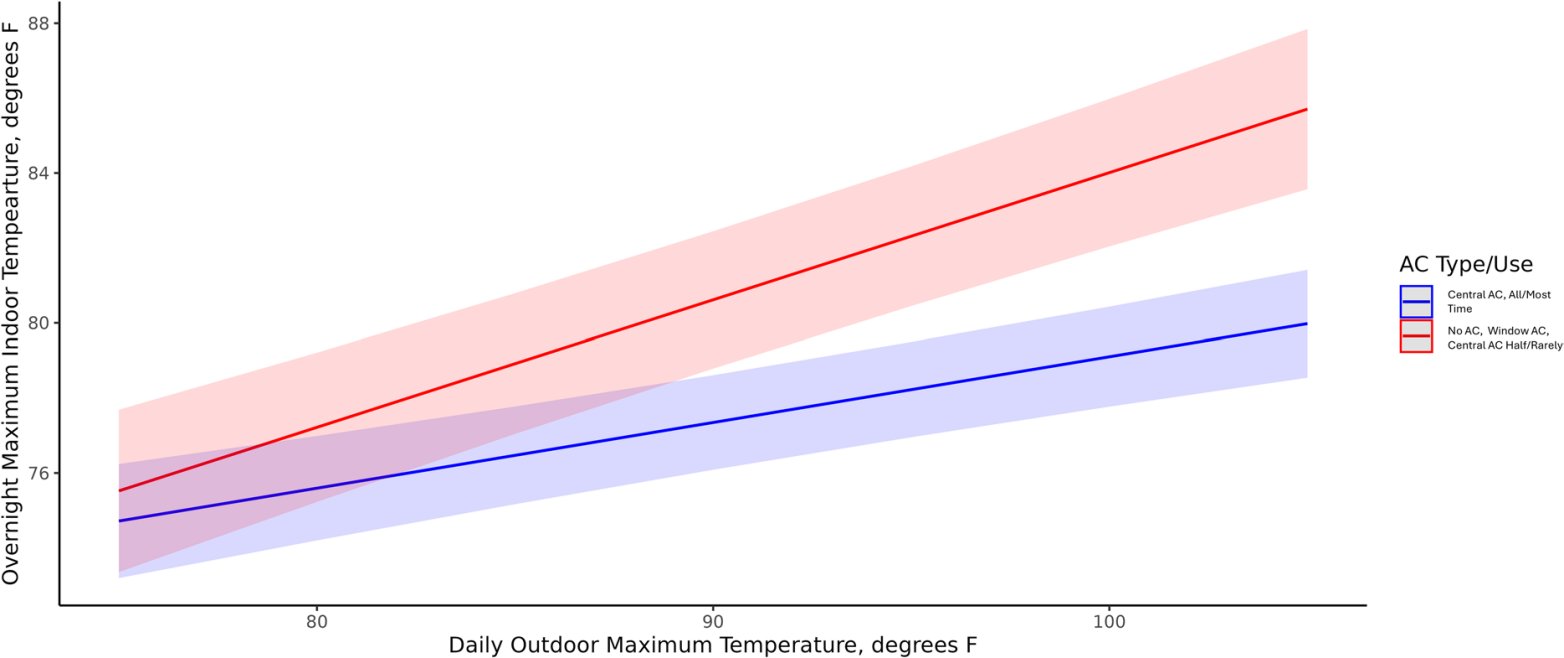
Predicted maximum indoor overnight temperature according to preceding day maximum outdoor temperature and air conditioning type and use, adjusted for energy costs, homeownership, and repeated measures (Shading indicates 95% confidence intervals)

In a model examining effect modification, there was a statistically significant interaction between daily maximum outdoor (preceding day) temperature and AC type/use (p-value for interaction = 0.007), indicating that there is a steeper rise in maximum overnight indoor temperature, for every unit increase in daily maximum outdoor temperature, in residences with no AC, window units, or those that are not running central AC all or most of the time during hot weather compared to residences that are running central AC all or most of the time during hot weather (Fig. 1). Sensitivity analysis examining predictors of daily *mean* overnight indoor temperature showed largely similar results with some predictors (e.g., energy costs and homeownership status) exhibiting more precise associations with mean overnight indoor temperature than with maximum overnight indoor temperature (Supplementary Table 3).

**Fig. 2:**
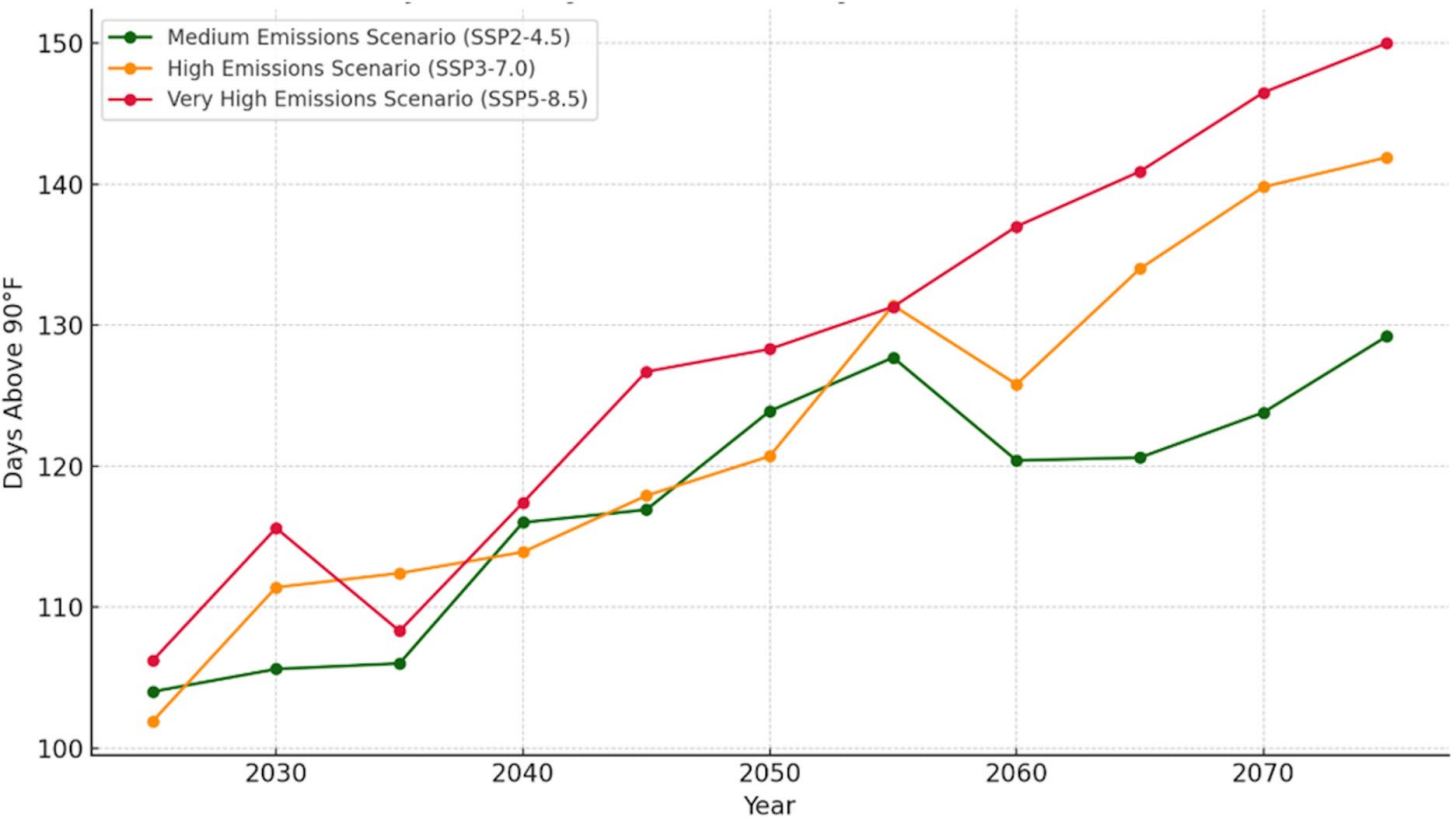
Projected days above 90° F between 2025–2075 in New Orleans by greenhouse gas emissions scenario (Data Source: Localized Constructed Analogs 2, LOCA2: https://loca.ucsd.edu/loca-version-2-for-north-america-ca-jan-2023/)

Among households with no AC, window units, or those not running central AC all or most of the time, the threshold for maintaining the Cooling Standard of 80 degrees Fahrenheit or lower was generally exceeded once outdoor daily maximum temperatures reached approximately 90 degrees Fahrenheit. Using this finding, we analyzed projections of the number of days that daily maximum temperatures will exceed 90 degrees Fahrenheit (MaxT > 90) in New Orleans between 2025 and 2075 (Fig. 2). We found that days with a high above 90 degrees Fahrenheit in New Orleans are projected to significantly increase under all three climate emissions scenarios, ranging from an additional 25 days (SSP2-4.5) to 50 days (SSP5-8.5) with a high above 90 degrees Fahrenheit per year by 2075.

**Fig. 3:**
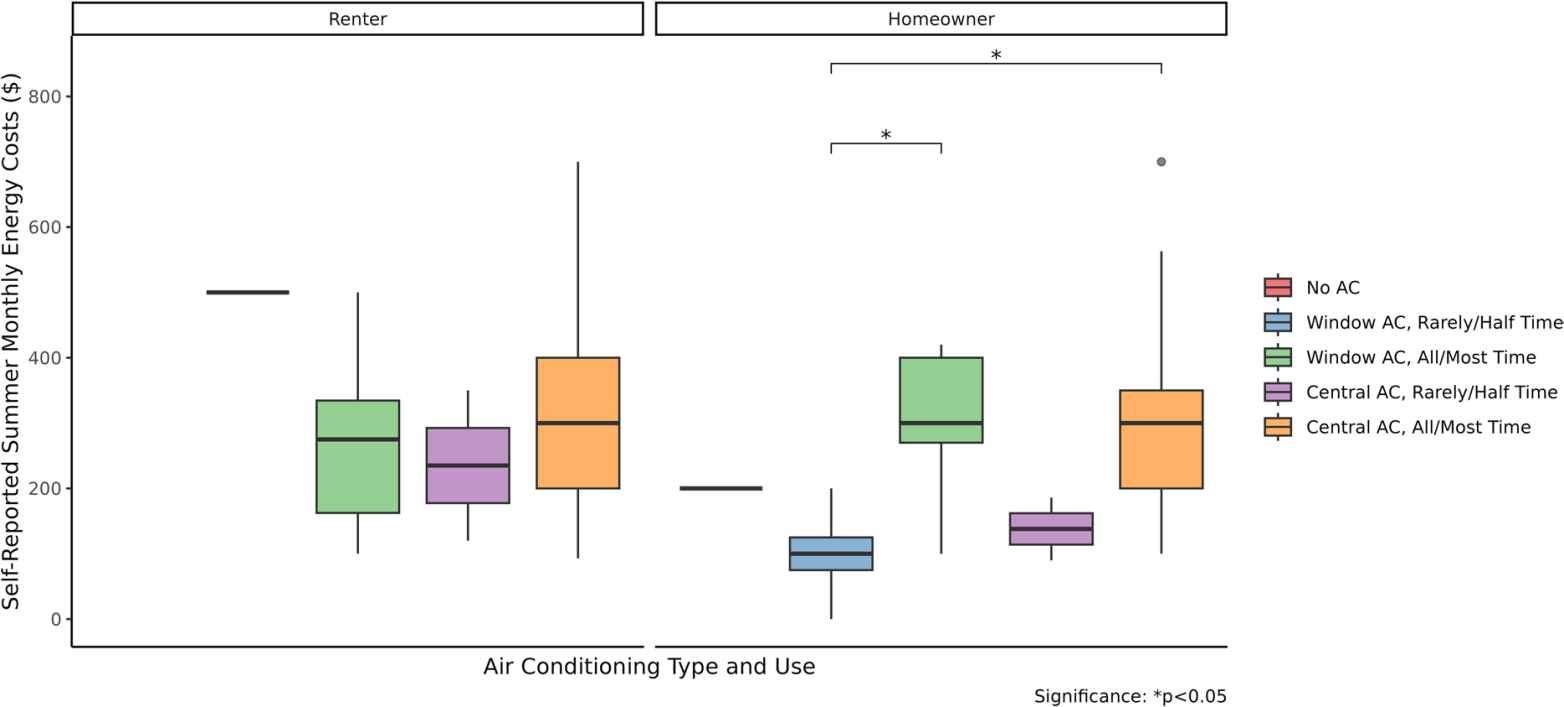
Distribution of self-reported summer monthly energy costs by air conditioning type and use across homeownership status

Because use and type of AC was a significant predictor of the overnight indoor thermal environment, we examined the distribution of participants’ monthly energy costs by use and type of AC. For renters in our sample, we did not find a statistically significant difference in mean self-reported summer monthly energy costs by use and type of AC. However, for homeowners, we found that both participants who reported using window AC or central AC all or most of the time during hot weather had a greater self-reported monthly summer energy costs (by approximately $200) than participants who reported using window AC’s rarely or less than half of the time during hot weather (Fig. 3).

## Discussion

Our study found that a combination of AC type and use (AC type/use), along with outdoor daily maximum temperature and time of season, were significant predictors of maximum indoor overnight temperature. Our results indicate that households without AC, those with central AC but not running it all or most of the time, and those with window units – even running all or most of the time – struggled to maintain 80 degrees Fahrenheit overnight once outdoor daily maximum temperatures exceeded 90 degrees Fahrenheit. This is notable given the New Orleans’s Healthy Homes Program requirement that:

Each rental housing unit shall have a cooling system in good working order that can safely maintain a maximum temperature of 80 degrees Fahrenheit in all bedrooms, measured at a point three feet above the floor and two feet from exterior walls [18].

Passage of this standard is a noteworthy and critical step to improve health and move heat adaptation forward but also raises questions about the health implications of these temperatures, which are not well known, as well as challenges to implementation. As the Healthy Homes Program was effective as of January 1, 2024, with phased implementation in 2024 and 2025, our results do not necessarily reflect conditions post-implementation.

Nevertheless, the fact that a large portion of our sample struggled to maintain 80 degrees Fahrenheit indoors once outdoor daily maximum temperatures exceeded 90 degrees Fahrenheit is particularly significant in light of climate change and rising global temperatures; as shown in Fig. 2, days with a high above 90 degrees Fahrenheit are projected to grow by up to 50%, to 150 days per year, by 2075 under the highest climate scenario (SSP5-8.5). This suggests that there will be an annual increase in cooling needs over time in order to maintain an 80 degrees Fahrenheit or lower threshold in residential buildings. This may exacerbate other needs, risks, and challenges identified in our study results.

While AC as a strong determinant of indoor thermal conditions is not a new finding [31, 35–37], this research demonstrates that more nuance and information than AC access alone, including information on AC type and use, is necessary for research on the indoor thermal environment, and, consequently, for research that attempts to link conditions of the indoor thermal environment to public health. Only a handful of small studies in the U.S. have gone beyond simply reporting AC prevalence to examine how variation in indoor thermal environments vary by AC type and use. For example, in a sample of 36 homes in NYC, central and ductless AC types were associated with the coolest indoor conditions, while homes with window and portable AC were significantly warmer [31]. Similarly, a study of 24 Boston residents found that although all participants had some form of AC, only 4 of the 17 with window or wall units reported that their AC units were adequate to keep them cool [35]. Our findings are consistent with these patterns, and they contribute to this body of evidence with data from the southeastern United States, a region of the country that is highly vulnerable to extreme weather and climate change.

Furthermore, prior research across the U.S. has found that Black, Hispanic, Indigenous, and multi-racial households have been shown to be between 1.2–1.7 times more likely to have window AC units than central AC [38] – a disparity linked to structural inequalities and historical marginalization. This is significant in light of our and the above study findings. Our ability to observe this pattern or, more broadly, household income and race/ethnicity as predictors of indoor temperatures, was limited due to the relative racial and socioeconomic homogeneity of our study population. However, this reflects an intentional focus of our study design: to center data collection within communities that have been shown to face higher extreme heat risks and challenges in order to capture more granular data that can be missed in broader, heterogeneous samples.

Our results also suggest differences between homeowners and renters. Homeowners in our participant group struggled more than renters to keep their homes cool during our study period despite a higher proportion of renters falling into the lowest income category (Supplementary Table 3). Approximately 62% of homeowners and 38% of renters were found in the highest quartile of mean daily overnight maximum temperatures, a distribution that was close to reversed in the lowest quartile. Further, our analysis of monthly energy cost data (Fig. 3) demonstrated that homeowners in our sample exhibited greater variability in typical AC use patterns as well as a greater sensitivity of summer monthly energy expenditures to differences in AC use patterns than renters. There are a number of plausible explanations for these findings, though we cannot rule out any from our data, alone. Due to a multitude of other financial obligations that come along with homeownership, such as upkeep, repair, and property taxes, low-income homeowners may be more likely to cut back on energy use than renters, even when necessary for cooling. The AC units, themselves, may not be comparable across homeowners and renters in terms of efficiency and performance. Finally, the amount of area required to cool is likely different across these two groups.

While renters are a uniquely vulnerable population when it comes to adaptive measures that require the installation of heating, ventilation, and AC machinery, our data highlight that homeowners, particularly low-income homeowners, can also face significant burden from heat. These findings are particularly relevant in light of the New Orleans cooling standard, which, like some other standards implemented in locales around the country, is applicable only for renter-occupied buildings and therefore does not address the burdens and risks of homeowners. Policymakers should consider low-income homeowners, in addition to renters, when designing heat protection strategies. Ultimately, the creation of indoor residential cooling standards is an important step towards healthier home environments, but implementation of these standards needs further study. There may be growing challenges to meeting standard thresholds, and the standards may not address all significant underlying issues associated with extreme heat exposure.

Understanding these challenges is essential for informing how cities and regions approach cooling and climate adaptation. While effective AC is a necessary strategy to maintain healthy temperatures and can be lifesaving during extreme heat events, growing AC use poses numerous climate and human health contradictions. This includes contradictions between climate adaptation and mitigation, as most AC contributes to greenhouse gas emissions and rising temperatures over time through the use of fossil fuels [39]. AC also places pressure on the electrical grid and can contribute to blackouts and rolling blackouts as energy demand soars during extreme heat events and exceeds grid capacity [40]. This undermines ability to use AC and puts human health at risk during critical periods, pointing to the need to develop novel solutions for indoor thermal comfort. For example, research has shown that natural ventilation combined with radiant cooling strategies can be used in every major climate zone and would result in significant energy savings while maintaining thermally comfortable indoor environments [41].

### Limitations and Future Research Recommendations

Limitations of this research included a lack of information on the hours of AC operation per participant, that temperatures were measured in one room of participants’ residential environment, and limited (participant-reported) information on the residential characteristics and structural integrity of participant homes. Without information on AC operation times for each residence, we cannot determine if the higher indoor overnight temperatures observed in households is due to the inherent performance of the AC unit or behavioral modifications that limit operation time of the unit. Though, it should be noted that prior literature has found that energy insecure households often practice energy limiting behavior and keep their homes at unhealthy temperatures [42, 43]. Actual AC use patterns may also explain the unexpected finding that portable fans are marginally associated with higher indoor overnight temperatures (due to residual confounding), as well as the greater univariate correlation observed between outdoor and indoor temperatures in Year 2 versus Year 1, since data collection in Year 2 extended later into the season and participants could be more likely to let indoor temperature drift with outdoor temperature. These differences may also be explained by a variety of other meteorological factors which will be investigated in future work. Energy bills were based on a one-time self-report, with validation of utility bills in a portion of participants. This study employed convenience sampling in neighborhoods and areas of New Orleans and is therefore not representative of New Orleans as a whole or the individual neighborhoods. Our study focused on populations and neighborhoods that have historically experienced high heat vulnerability, and thus, was relatively homogenous with respect to racial and socioeconomic characteristics. This, along with the fact that some vulnerability characteristics are correlated within populations, may have limited our ability to detect certain predictors of indoor temperatures that studies with more heterogeneous samples observe.

Furthermore, although we centered a portion of our analysis and discussion around the cooling standard upper limit of 80° Fahrenheit, the health implications of these temperatures are not well known, and this study was not designed to test the effectiveness of this standard. For example, existing research has found that temperature limits of 80° Fahrenheit may be too high to be adequately protective [44]. More research is needed to better understand healthy upper ambient air temperature and humidity thresholds in indoor environments.

Overall, there is a need for further research into cooling behaviors, adaptive capacity, and thermal conditions, particularly among heat-vulnerable populations. More research on the effectiveness of interventions that can increase AC performance and efficiency, such as structural improvements to residential buildings that enhance insulation, as well as programs that can provide financial assistance to renters and homeowners so that they are able to run AC during hot weather, is also needed.

## Conclusions

In a sample of owner- and renter-occupied units from residents of multiple low-income, predominantly Black neighborhoods across two wards in New Orleans, Louisiana, we observed that daily maximum outdoor temperature, AC type and use, and timing of season were significant predictors of maximum overnight indoor temperature. Households with no AC, window units, or that were not running central AC all or most of the time during very hot weather had a lower likelihood of maintaining an indoor overnight temperature below 80° F, and this inability was exacerbated as outdoor temperatures rose. Homeownership, compared to renting, was associated with higher overnight indoor temperatures. Homeowners in our sample also exhibited greater variability in typical AC use patterns as well as a greater sensitivity of summer monthly energy expenditures to AC use patterns than renters. Taken together, this could indicate that homeowners from our sample were more likely to practice energy limiting behavior, an area that deserves further attention when considering heat adaptation for low-income populations.

This paper contributes to limited literature on indoor thermal environments and housing characteristics in the context of climate change and health. It underscores the importance of housing in relation to climate change and structural determinants of health. The study’s focus on the Southeastern US is of particular note given the high climate change vulnerability of this area and high levels of AC penetration. It highlights the need to think holistically about protections against extreme heat in the residential environment, which may suggest the need for structural upgrades and more comprehensive utility assistance for both homeowners and renters in addition to indoor residential cooling standards. This paper further underscores the importance of analyzing the extent to which extreme heat days will rise in the near future, which contextualizes current challenges and heightens the urgency to understand and address residential thermal exposure and heat risk.

Overall, our results suggest a challenge of maintaining cooling standard requirements in the present, particularly for households not running central AC all or most of the time, with a growing challenge in the future as high temperature events become more frequent. Despite its potential for protective effects, contradictions posed by growing global AC use suggest that while AC is necessary, it alone is not a viable long-term, systematic adaptation to extreme heat. In addition, our results suggest that access to AC, alone, does not always provide protective cooling effects; rather, type and use of AC are important factors in residential cooling and may also contribute to resident energy burden. As more cities across the country and world consider indoor cooling standards, more research is needed to understand healthy temperature thresholds as well as equitable implementation needs and challenges.

## Supplementary Information

Below is the link to the electronic supplementary material.

ESM 1 (DOCX 290 KB )

## Data Availability

The data analyzed in this study are not publicly available due to human subjects privacy protections.
