# Supplementary material for: Indoor Thermal Environments, Cooling Access, and Energy Burden in New Orleans, LA: Challenges and Opportunities for Heat Adaptation
Source: J Urban Health. 2026 Jun 10;103(3):469–83. doi: 10.1007/s11524-026-01096-w (PMC13315058; doi:10.1007/s11524-026-01096-w)
Supplement: Supplementary file 1 — (DOCX 290 KB ) [file 11524_2026_1096_MOESM1_ESM.docx]

**Supplementary Material**

**Title:** Indoor Thermal Environments, Cooling Access, and Energy Burden in New Orleans, LA: Challenges and Opportunities for Heat Adaptation

**Authors**: Lena Easton-Calabria^1,2^, Caroline Reed^3^, Teague Ruder^1^, Jordan Mychal^3^, Jacopo Scazzosi^3^, Ramya Chari^4^, Brian Vant-Hull^5^, Julia Kumari Drapkin^3^, Jaime Madrigano^6^

**Affiliations:**

^1^ RAND, Arlington, VA, USA

^2^ University of Oxford, Oxford, UK

^3^ ISeeChange, Inc., New Orleans, LA, USA

^4^ RAND, Boston, MA, USA

^5^ The City College of New York, New York, NY, USA

^6^ Johns Hopkins Bloomberg School of Public Health, Baltimore, MD, USA

**Corresponding Author:**

Jaime Madrigano

[jmadrig4@jhu.edu](mailto:jmadrig4@jhu.edu)

Johns Hopkins Bloomberg School of Public Health

615 N. Wolfe Street

Baltimore, MD 21205

| **Supplementary Table 1. Person-Time Contribution According to Population and Residential Characteristics by Quartiles of Mean Daily Overnight Indoor Maximum Temperature** | | | | | |
| --- | --- | --- | --- | --- | --- |
|  |  |  |  |  |  |
| Characteristic, person-days (N, %) | Overall | Q1 (65.9-75 °F) | Q2 (75.1-78 °F) | Q3 (78.1-81.8 °F) | Q4 (81.9-101.0 °F) |
| Race/Ethnicity |  |  |  |  |  |
| Black or African American | 1374, 85.7% | 346, 86.3% | 337, 84% | 342, 85.5% | 349, 87.2% |
| Hispanic/Latino | 11, 0.7% | 0 | 0 | 0 | 11, 2.8% |
| American Indian or Alaska Native | 4, 0.2% | 2, 0.5% | 1, 0.2% | 1, 0.2% | 1, 0.2% |
| White | 143, 8.9% | 34, 8.5% | 38, 9.5% | 36, 9% | 35, 8.8% |
| Multiracial | 39, 2.5% | 9, 2.2% | 21, 5.2% | 7, 1.8% | 2, 0.5% |
| Other | 2, 0.1% | 2, 0.5% | 0 | 0 | 0 |
| Prefer not to say | 29, 1.8% | 8, 2% | 5, 1.2% | 14, 3.5% | 2, 0.5% |
| Gender |  |  |  |  |  |
| Woman | 1166, 72.7% | 337, 84.2% | 315, 78.6% | 260, 66.2% | 254, 64.8% |
| Man | 375, 23.4% | 42, 10.5% | 69, 17.2% | 127, 32.3% | 137, 34.9% |
| Non-binary | 14, 0.9% | 0,0% | 9, 2.2% | 4, 1% | 1, 0.3% |
| Prefer not to say | 31, 1.9% | 21, 5.2% | 8, 2% | 2, 0.5% | 0.0% |
| Annual Income |  |  |  |  |  |
| $0-$14,999 | 677, 42.4% | 218, 54.4% | 149, 37.2% | 164, 41% | 146, 36.5% |
| $15,000-$44,999 | 516, 32.2% | 121, 30.2% | 124, 30.9% | 121, 30.2% | 150, 37.5% |
| $45,000-$89,999 | 252, 15.7% | 28, 7% | 102, 25.4% | 66, 16.5% | 56, 14% |
| $90,000+ | 30, 1.9% | 1, 0.2% | 12, 3% | 4, 1% | 13, 3.2% |
| Prefer not to say | 127, 7.9% | 33, 8.2% | 14, 3.5% | 45, 11.2% | 35, 8.8% |
| Homeownership Status |  |  |  |  |  |
| Rental | 812, 50.6% | 257, 64.1% | 222, 55.4% | 180, 45% | 153, 38.2% |
| Own/family owns home | 790, 49.3% | 144, 35.9% | 179, 44.6% | 220, 55% | 247, 61.8% |
| Home Type |  |  |  |  |  |
| Single-family home | 997, 62.2% | 240, 59.9% | 255, 63.6% | 254, 63.5% | 248, 62% |
| Double-family home (New Orleans shotgun or Camelback) | 365, 22.8% | 108, 26.9% | 80, 20% | 88, 22% | 89, 22.2% |
| Apartment/multi-unit housing | 156, 9.7% | 31, 7.7% | 53, 13.2% | 32, 8% | 40, 10% |
| Other | 84, 5.2% | 22, 5.5% | 13, 3.2% | 26, 6.5% | 23, 5.8% |
| Home Material |  |  |  |  |  |
| Wood | 864, 53.9 | 210, 52.4% | 180, 44.9% | 222, 55.5% | 252, 63% |
| Concrete | 102, 6.4% | 43, 10.7% | 30, 7.5% | 18, 4.5% | 11, 2.8% |
| Brick | 81, 5% | 15, 3.7% | 18, 4.5% | 16, 4% | 32, 8% |
| Multiple/unknown | 555, 34.6% | 133, 33.2% | 173, 43.1% | 144, 36% | 105, 26.2% |
| AC Type |  |  |  |  |  |
| Central | 1212, 75.6% | 355, 88.5% | 354, 88.3% | 295, 73.8% | 208, 52% |
| Window | 363, 1.7% | 46, 11.5% | 45, 11.2% | 96, 24% | 176, 44% |
| None | 27, 1.7% | 0 | 2, 0.5% | 9, 2.2% | 16, 4% |
| Self-report trees shade home |  |  |  |  |  |
| Yes | 281, 17.5% | 45, 11.2% | 68, 17% | 72, 18% | 96, 24% |
| No | 1162, 72.4% | 333, 83% | 282, 70.3% | 282, 70.5% | 265, 66.2% |
| Partially | 159, 9.9% | 23, 5.7% | 51, 12.7% | 46, 11.5% | 39, 9.8% |
| Use of Fans |  |  |  |  |  |
| None | 206, 12.8% | 99, 25.1% | 50, 12.7% | 36, 9.2% | 18, 4.6% |
| Ceiling | 610, 38% | 154, 39.1% | 171, 43.4% | 135, 34.6% | 138, 35.6% |
| Multiple | 479, 29.9% | 87, 22.1% | 135, 34.3% | 127, 32.6% | 114, 29.4% |
| Portable | 309, 19.3% | 54, 13.7% | 38, 9.6% | 92, 23.6% | 118, 30.4% |
| Use of Other Means of Cooling |  |  |  |  |  |
| Multiple | 659, 41.1% | 146, 37.1% | 180, 45.7% | 155, 39.7% | 159, 41% |
| Blinds | 660, 41.1% | 143, 36.3% | 163, 41.4% | 167, 42.8% | 174, 44.8% |
| Open doors | 82, 5.1% | 31, 7.9% | 19, 4.8% | 9, 2.3% | 21, 5.4% |
| Other items | 178, 11.1% | 66, 16.8% | 26, 6.6% | 51, 13.1% | 32, 8.2% |
| Open window | 25, 1.6% | 8, 2% | 6, 1.5% | 8, 2.1% | 2, 0.5% |
| Floor that Participant Sleeps on |  |  |  |  |  |
| Zero | 123, 7.7% | 11, 2.8% | 24, 6.1% | 30, 7.7% | 58, 14.9% |
| One | 1194, 74.4% | 318, 80.7% | 306, 77.7% | 271, 69.5% | 270, 69.6% |
| Two | 269, 16.8% | 58, 14.7% | 59, 15% | 83, 21.3% | 60, 15.5% |
| Three | 18, 1.1% | 7, 1.8% | 5, 1.3% | 6, 1.5% | 0 |
| Study Year |  |  |  |  |  |
| One | 971, 60.5% | 256, 63.8% | 209, 52.1% | 220, 55% | 286, 71.5% |
| Two | 631, 39.3% | 145, 36.2% | 192, 47.9% | 180, 45% | 114, 28.5% |
| Month |  |  |  |  |  |
| July | 174, 11% | 58, 14% | 34, 8.5% | 32, 8.0% | 50, 13% |
| August | 596, 37% | 114, 28% | 161, 40% | 117, 44% | 144, 36% |
| September | 490, 31% | 164, 41% | 95, 24% | 126, 32% | 105, 26% |
| October | 342, 21% | 65, 16% | 111, 28% | 65, 16% | 101, 25% |
| Month-Year |  |  |  |  |  |
| July 2023 | 174, 11% | 58, 14% | 34, 8.5% | 32, 8.0% | 50, 13% |
| August 2023 | 319, 20% | 82, 20% | 75, 19% | 86, 22% | 76, 19% |
| September 2023 | 216, 13% | 61, 15% | 36, 9.0% | 52, 13% | 67, 17% |
| October 2023 | 262, 16% | 55, 14% | 64, 16% | 50, 13% | 93, 23% |
| August 2024 | 277, 17% | 32, 8.0% | 86, 21% | 91, 23% | 68, 17% |
| September 2024 | 274, 17% | 103, 26% | 59, 15% | 74, 19% | 38, 9.5% |
| October 2024 | 80, 5.0% | 10, 2.5% | 47, 12% | 15, 3.8% | 8, 2.0% |
| Outdoor Maximum Temperature  (Mean, SD) | 92.1 (5.4) | 90.9 (6.0) | 92.1 (5.2) | 92.4 (5.5) | 92.9 (4.9) |

| **Supplementary Table 2. Mean difference (and 95% confidence interval) in daily overnight mean indoor temperature associated with a one-unit increase of or in comparison to a reference value for independent variables.*** | | | | | | | | |
| --- | --- | --- | --- | --- | --- | --- | --- | --- |
|  | | | | | | | | |
|  | Estimate | 95% CI | | p-value |  |  | |  |
|  |  |  |  |  |  |  |  |  |
| Daily maximum outdoor temperature (per 1°F) | 0.11 | 0.07 | 0.16 | <0.0001 |  |  |  |  |
| Air Conditioning Type and Use |  |  |  |  |  |  |  |  |
| Central AC, All/Most Time | Reference |  |  |  |  |  |  |  |
| No AC, Any Window AC, Central AC Half Time/Rarely | 2.93 | 0.76 | 5.10 | 0.009 |  |  |  |  |
| Race |  |  |  |  |  |  |  |  |
| All other races | Reference |  |  |  |  |  |  |  |
| Black race | -0.87 | -4.55 | 2.81 | 0.639 |  |  |  |  |
| Gender |  |  |  |  |  |  |  |  |
| Female | Reference |  |  |  |  |  |  |  |
| Male | 1.39 | -0.82 | 3.61 | 0.215 |  |  |  |  |
| Non-binary | -3.92 | -14.59 | 6.76 | 0.467 |  |  |  |  |
| Age (per 1 year) | 0.00 | -0.07 | 0.07 | 0.963 |  |  |  |  |
| Household Income |  |  |  |  |  |  |  |  |
| $0-$14,999 | Reference |  |  |  |  |  |  |  |
| $15,000 -$44,999 | -0.10 | -2.24 | 2.04 | 0.926 |  |  |  |  |
| $45,000 - $89,999 | -3.06 | -6.26 | 0.13 | 0.060 |  |  |  |  |
| $90,000 + | 0.07 | -6.56 | 6.69 | 0.984 |  |  |  |  |
| Refused to answer | -0.84 | -4.45 | 2.78 | 0.646 |  |  |  |  |
| Home Type |  |  |  |  |  |  |  |  |
| Single | Reference |  |  |  |  |  |  |  |
| Apartment/multi unit | 2.54 | -0.40 | 5.49 | 0.089 |  |  |  |  |
| Duplex/Double | -0.48 | -2.91 | 1.94 | 0.692 |  |  |  |  |
| Other | 0.52 | -3.92 | 4.96 | 0.816 |  |  |  |  |
| Home Material |  |  |  |  |  |  |  |  |
| Brick | Reference |  |  |  |  |  |  |  |
| Concrete | -2.30 | -7.11 | 2.51 | 0.344 |  |  |  |  |
| Multiple/Unknown | -0.12 | -4.06 | 3.83 | 0.953 |  |  |  |  |
| Wood | -1.17 | -4.83 | 2.48 | 0.524 |  |  |  |  |
| Study Cohort |  |  |  |  |  |  |  |  |
| 1 | Reference |  |  |  |  |  |  |  |
| 2 | -0.13 | -0.62 | 0.36 | 0.609 |  |  |  |  |
| 3 | 1.67 | -0.63 | 3.97 | 0.153 |  |  |  |  |
| 4 | -0.40 | -3.15 | 2.34 | 0.771 |  |  |  |  |
| 5 | -2.43 | -5.42 | 0.56 | 0.110 |  |  |  |  |
| 6 | -1.65 | -5.56 | 2.26 | 0.403 |  |  |  |  |
| Self-reported energy costs (per $) | -0.01 | -0.02 | 0.00 | 0.009 |  |  |  |  |
| Self-reported trees provide shade to residence |  |  |  |  |  |  |  |  |
| No | Reference |  |  |  |  |  |  |  |
| Yes | 1.50 | -0.51 | 3.51 | 0.141 |  |  |  |  |
| Homeownership |  |  |  |  |  |  |  |  |
| Renter | Reference |  |  |  |  |  |  |  |
| Owns home | 2.34 | -0.04 | 4.73 | 0.054 |  |  |  |  |
| Use of fans |  |  |  |  |  |  |  |  |
| None | Reference |  |  |  |  |  |  |  |
| Ceiling | 0.93 | -2.51 | 4.36 | 0.594 |  |  |  |  |
| Multiple | 2.38 | -1.35 | 6.10 | 0.207 |  |  |  |  |
| Portable | 3.12 | -0.26 | 6.49 | 0.070 |  |  |  |  |
| Use of other means of cooling |  |  |  |  |  |  |  |  |
| Multiple | Reference |  |  |  |  |  |  |  |
| Blinds | 0.64 | -1.43 | 2.71 | 0.542 |  |  |  |  |
| Open doors | -0.60 | -5.42 | 4.21 | 0.804 |  |  |  |  |
| Other items | -0.83 | -3.99 | 2.32 | 0.601 |  |  |  |  |
| Open window | 1.19 | -5.04 | 7.42 | 0.704 |  |  |  |  |
| Floor that participant sleeps on (per level) |  |  |  |  |  |  |  |  |
|  | |  |  |  |  |  |  |  |
| * Models included a random intercept for participant; N=109, with 1,532 person-days of observation. Three participants were missing information on gender and 2 participants were missing information on homeownership status. Two person-days were excluded because of missing outdoor temperature values. | | | | | | | | |

**Supplementary Table 3**. Income Distribution of Homeowners Versus Renters

| Income | Homeowner (%) | Renter (%) |
| --- | --- | --- |
| $0-$14,999 | 24.2 | 63.1 |
| $15,000-$44,999 | 32.3 | 27.7 |
| $45,000-$89,999 | 25.8 | 4.6 |
| $90,000+ | 3.2 | 0 |
| Refused to answer | 14.5 | 4.6 |

**Supplementary Fig. 1** Correlation Between Maximum Overnight Indoor Temperature and Daily Outdoor Maximum Temperature, By Study Cohort


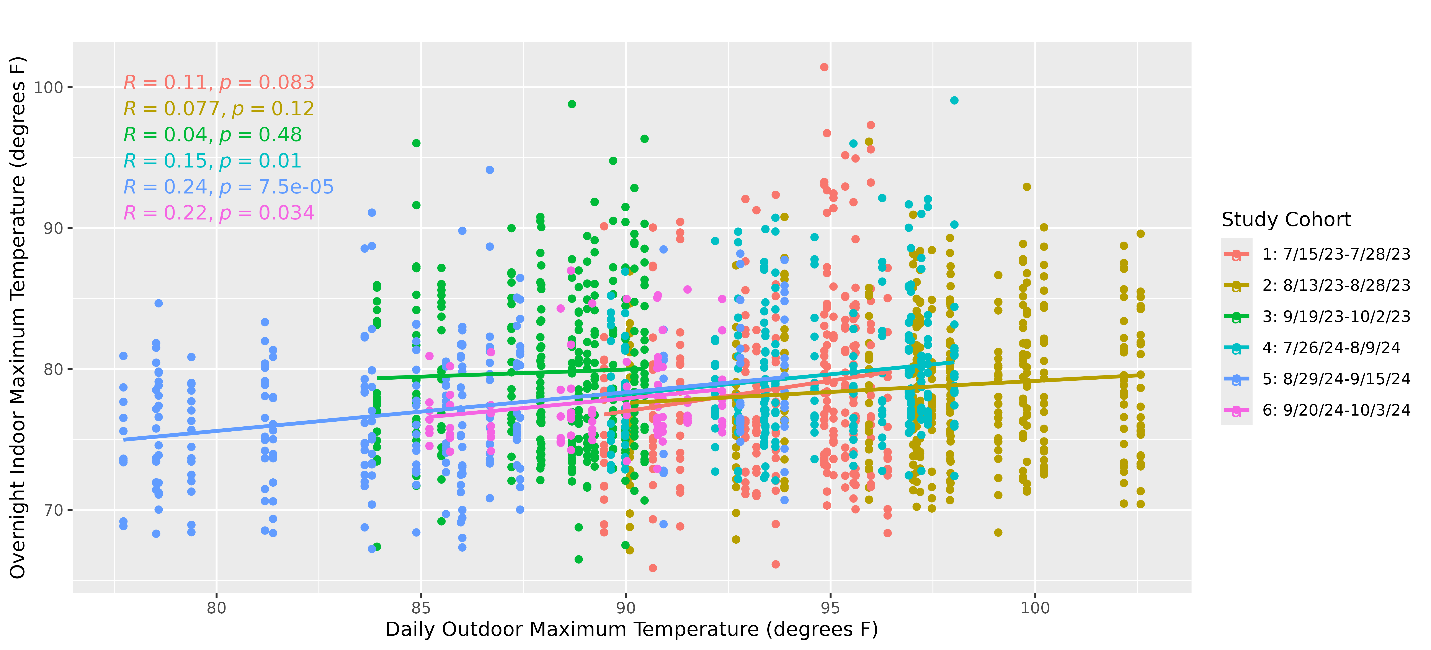


**Supplementary Fig. 2** Study Intake Survey

Confirm eligibility information:

1. Name:
2. Age [verify that participant is at least 18 years; otherwise exclude]:
3. Home address:

[If answers differ from phone screening and participant is ineligible, display reads: I’m sorry, you are not eligible to participate in the study at this time. If eligible, proceed to informed consent.]

Once informed consent documented, proceed:

This survey will take approximately 30 minutes to complete. Please answer each question to the best of your ability.

1. Email address:
2. Phone Number (cell):
3. Study ID:
4. How would you describe yourself? (Check all that apply)
   1. American Indian or Alaska Native
   2. Asian
   3. Black or African American
   4. Native Hawaiian or Other Pacific Islander
   5. Hispanic, Latino/Latinx, or of Spanish origin
   6. White
   7. Other: __________
5. What is your native language?
   1. English
   2. Spanish
   3. Vietnamese
   4. French
   5. Other: ___________
6. Gender identity (check all that apply)
   1. Woman
   2. Man
   3. Non-binary
   4. Transgender
   5. Cisgender
   6. Intersex
   7. I prefer not to say
   8. Other: _____________
7. Sex assigned at birth
   1. Female
   2. Male
   3. Intersex
   4. I prefer not to say
8. What is the highest degree or level of school you have completed? (If you’re currently enrolled in school, please indicate the highest degree you have *received.*)
   1. Less than a high school diploma
   2. High school degree or equivalent (e.g. GED)
   3. Some college, no degree
   4. Associate degree (e.g. AA, AS)
   5. Bachelor’s degree (e.g. BA, BS)
   6. Master’s degree (e.g. MA, MS, MEd)
   7. Professional degree (e.g. MD, DDS, DVM)
   8. Doctorate (e.g. PhD, EdD)
9. What is your current employment status?
   1. Employed full time (40 or more hours per week)
   2. Employed part time (up to 39 hours per week)
   3. Unemployed and currently looking for work
   4. Unemployed and not currently looking for work
   5. Student
   6. Retired
   7. Homemaker
   8. Self-employed
   9. Unable to work due to a disability
   10. Unable to work due to another reason
10. If currently employed, what is your occupation?
11. What is your main method of transportation?
    1. Public transportation
    2. Personal car
    3. Bicycle
    4. Walking
    5. I prefer not to say
12. Are you currently living…
    1. In a rental house or apartment
    2. In a rental house or apartment with public assistance (Section 8 or subsidized housing)
    3. In a home you or your family owns
    4. In a home of a friend or acquaintance
    5. With homelessness / unhoused
13. If renting or in public assistance housing, is your landlord attentive/do they respond promptly if you bring up an issue?
    1. Yes
    2. No
    3. Other: ___________________________
14. Including yourself, how many people are living in your household? ______
15. What is your annual household income?
    1. $0-15,000
    2. $15,000-30,000
    3. $30,000-45,000
    4. $45,000-60,000
    5. $60,000-75,000
    6. $75,000-90,000
    7. $90,000+
    8. I prefer not to say
16. Do you consider yourself a high, middle or low income home?
    1. High income
    2. Middle income
    3. Low income
17. Have you ever been told by a doctor, or other health professional, that you have any of the following conditions? (Check all that apply)

|  | **YES** | **NO** | **DON’T KNOW / NOT SURE** | **REFUSED TO ANSWER** |
| --- | --- | --- | --- | --- |
| Heart disease |  |  |  |  |
| Kidney disease |  |  |  |  |
| Diabetes or high blood sugar |  |  |  |  |
| High blood pressure or hypertension |  |  |  |  |
| High cholesterol |  |  |  |  |
| Arthritis |  |  |  |  |
| Cancer |  |  |  |  |
| Asthma |  |  |  |  |
| COPD (Chronic Obstructive Pulmonary Disease) |  |  |  |  |
| A mental health condition |  |  |  |  |

1. Are you currently pregnant?
   1. Yes
   2. No
   3. Unsure

Heat Experience Questions

1. Do you live in….
   1. A single-family home
   2. A double-family home (New Orleans shotgun or Camelback)
   3. Multi-unit housing (single story)
   4. Apartment complex (multiple floors)
   5. Other: _________________
2. If you live in an apartment building with multiple floors, what floor do you live on?
   1. _____________________
3. How many total floors are in your building or home?
   1. _____________________
4. What floor of your house/apartment is the room you sleep in?
   1. _____________________
5. What kind of material is your house/apartment made out of?
   1. Wood
   2. Brick
   3. Concrete
   4. Other: ________________
   5. I don’t know
6. What kind of air conditioning do you have?
   1. Central air conditioning
   2. Window unit air conditioners
   3. None
7. Does the room you sleep in have AC?
   1. Yes
   2. No
8. [If Q5 = a or b] How often do you use air conditioning when you are at home during hot or very hot weather?
9. Always
10. Most of the time
11. About half the time
12. Rarely
13. Never
14. [If ‘rarely’ or ‘never’ to Q6] What is the main reason for not turning on your air conditioner during very hot weather? (Select the best answer)
    1. I do not usually feel hot.
    2. I am concerned that the electric bill would be too high.
    3. I want to conserve energy.
    4. My air conditioner is broken/not functioning properly.
    5. I do not like air conditioning.
    6. My air conditioner makes too much noise.
    7. My air conditioning negatively impacts my health (allergies/respiratory impacts).
    8. I am not able to control my thermostat.
    9. I do not use air conditioning for some other reason than those listed above.
15. Do you use fans in your home?
    1. Yes
    2. No
16. [If yes to Q8] What kind of fans do you have? (select all that apply)
    1. Portable fans
    2. Ceiling fans
    3. Attic fans
17. Do you use a dehumidifier in your home?
    1. Yes
    2. No
18. What additional methods do you use to adjust or control your home’s temperature? (Check all that apply)
    1. Adjust window blinds or shades
    2. Adjust operable window
    3. Adjust doors to exterior spaces (porch, backyard, etc.)
    4. Other items to block or reflect light or air from windows or doors (aluminum foil, cloth, weather strip, etc.)
    5. Other: ______________
19. What are your monthly energy costs (on average) during the summer?
    1. _______________
20. During hot or very hot weather, how satisfied are you with the temperature in your house/apartment?
    1. Very satisfied
    2. Somewhat satisfied
    3. Slightly satisfied
    4. Neutral
    5. Slightly satisfied
    6. Somewhat dissatisfied
    7. Very dissatisfied
21. [If e, f, or g to Q13] What is the source of you being dissatisfied or less than fully satisfied? (Check all that apply)
    1. Don’t have AC
    2. AC is inadequate or doesn’t function properly
    3. AC is too expensive
    4. Humidity too high (damp)
    5. Incoming sun
    6. Hot surrounding surfaces (floor, ceiling, walls or windows)
    7. Deficient window (not operable)
    8. Holes or cracks in your walls, floors, or windows
    9. Other: ____________
22. What are your main concerns about heat and humidity in your home?
    1. Keeping the home cool during high heat periods [temperature control]
    2. High electricity bills because of AC or fan use [energy costs]
    3. The health effects of heat including effects on existing chronic conditions [health effects]
    4. Discomfort in daily activities such as sleeping, working, or other activities because of heat or humidity [comfort]
    5. Indoor air quality and what we breathe in during hot and humid conditions [air quality]
    6. Heat and humidity causing damage to the home such as mold or structural issues [home maintenance]
    7. The larger environmental consequences of high energy usage such as greater outdoor air pollution or worsening climate change effects [environmental impacts]
    8. No way to control heat or cool off due to lack of AC or fans or AC or fans that don’t work [equipment]
23. If it is hot outside/in your home, can you walk to…
    1. A shaded area/park?
    2. A pool?
    3. An air conditioned public space/cooling center?
    4. Other shaded or cooling space?
    5. None
24. Do you enjoy/feel comfortable utilizing this park/pool/cooling center/space when it is hot outside?
    1. Yes
    2. No
25. Are there trees that provide shade to your home/apartment?
    1. Yes
    2. No
    3. Partially
26. Does your home/apartment get direct sunlight? (Check all that apply)
    1. In the Morning
    2. In the Early Afternoon
    3. In the Late Afternoon
    4. My home/apartment does not get direct sunlight
27. In which direction do your windows face? (Check all that apply)
    1. East
    2. West
    3. North
    4. South
    5. Northwest Corner
    6. Northeast Corner
    7. Southwest Corner
    8. Southeast Corner
    9. I don’t know
28. Have you received energy bill assistance in the last year?
    1. Yes
    2. No
    3. I’m not sure
29. Where or how have you received energy bill assistance?
    1. Low Income Home Energy Assistance Program (LIHEA)
    2. Church
    3. Other

Additional questions

1. How concerned are you about the potential heat impacts of losing power or AC?
   1. I haven’t thought about this
   2. Not concerned
   3. Somewhat concerned
   4. Concerned
   5. Very concerned
2. Have you lost power from any of the following in the last two years? Check all that apply.
   1. A storm/hurricane
   2. Electricity shut off due to inability to pay bills
   3. Maintenance shutoff
   4. Other (Please describe)
   5. None
3. How often have you lost power or AC in the last 2 years
   1. Never
   2. Once in the past 2 years
   3. Once per year
   4. Multiple times a year

1. When this occurred, how comfortable was the temperature in your house/apartment?
   1. Very comfortable
   2. Somewhat comfortable
   3. Neutral
   4. Slightly uncomfortable
   5. Very uncomfortable

1. When this occurred, did you stay in your home?
   1. Yes
   2. No, went to another home in New Orleans with AC
   3. No, went to a cooling center, public library, recreational center, or other public space
   4. No, went to a hotel
   5. No, left the city
   6. Other: _________________
2. Have you directly experienced a natural disaster or severe hurricane (such as Hurricanes Ida, Zeta, Isaac, Katrina, or other extreme storm)?
   1. Yes
   2. No
   3. Don’t know / can’t remember
   4. Prefer not to answer
3. During or following the event(s) referred to in the previous question (Q5), did you experience any of the following? (Choose all that apply)
   1. Being forced to leave your home for more than a month
   2. Emergency rescue
   3. Financial hardship
   4. Destruction of property
   5. Loss of a loved one
   6. Health impacts to you or your family
   7. Power outage impacts (not functioning A/C, lack of communication tools, other disruptions to daily life)
   8. Don’t know / can’t remember
   9. Prefer not to answer
4. How physically uncomfortable or difficult of an experience was the event(s) referred to in Q5 for you, on a scale of 1-5, with 5 being the most difficult and 1 being the least difficult?
   1. 1
   2. 2
   3. 3
   4. 4
   5. 5
   6. Prefer not to answer
5. How emotionally uncomfortable or difficult of an experience was the event(s) referred to in Q5, on a scale of 1-5, with 5 being the most difficult and 1 being the least difficult?
   1. 1
   2. 2
   3. 3
   4. 4
   5. 5
   6. Prefer not to answer

Thank you! Please hand the tablet back to Caroline or Jordan.

[The following questions will be asked verbally]

1. Using the information measured by the sensor, we can create a personalized report on the heat and humidity patterns in your home. Would you like to receive such a report for your home?

- Yes
- No

1. Would you be interested in knowing how your experiences with heat compare to others in the study?

- Yes
- No

1. Based on the results of this study and the information you and other participants provide, we will gain knowledge about a lot of different heat-related topics. For example, we will learn more about the health implications of heat and humidity, how to improve the heat and energy efficiency of homes like yours, and how results could be used by researchers, policy makers, advocates, or others to help improve heat conditions in homes. Are there other kinds of information you would find useful to learn from the study?

Write in: __________________

1. Would you like to receive communications about study progress and results?

- Yes
- No

1. Are you familiar with Energy Smart or other energy efficiency programs for Orleans residents?

- Yes
- No
